# Supplementary material for: Evaluation of 3D Human Intestinal Organoids as a Platform for EV-A71 Antiviral Drug Discovery
Source: Cells. 2023 Apr 12;12(8):1138. doi: 10.3390/cells12081138 (PMC10136548; doi:10.3390/cells12081138)

Evaluation of 3D human intestinal organoids as a platform for EV-A71 antiviral drug discovery

Fatma Masmoudi, Nanci Santos-Ferreira, Dasja Pajkrt, Katja C. Wolthers, Jeroen DeGroot, Maria L.H. Vlaming, Joana Rocha-Pereira, and Ludovico Buti

SUPPLEMENTARY MATERIALS

Supplemental Table S1. TaqMan gene expression assay
Supplemental Figure S1. Immunofluorescence images of differentiated HIOs.
Supplemental Figure S2. EV-A71 replication kinetics in RD cells
Supplemental Figure S3. Brightfield images of HIOs and RD cells.
Supplemental Figure S4. Immunofluorescence images of HIOs.
Supplemental Figure S5. Immunofluorescence images of RD cells.

Supplemental Table S1. TaqMan gene expression assay

Gene	Description I	Description II	Cat. Number
EEF1A1	Eukaryotic Translation Elongation Factor 1 Alpha 1	Housekeeping gene	Hs04987073_g1
LGR5	Leucine-rich repeat-containing G-protein coupled receptor 5	Stem cells	Hs00969429_m1
VIL1	Villin 1 protein	Enterocytes	Hs01031723_m1
CHGA	Chromogranin A	Enteroendocrine	Hs00900371_m1
LYZ	Lysozyme	Paneth cells	Hs00426232_m1

Supplemental Figure S1. Immunofluorescence images of differentiated HIOs. DAPI (blue) for nucleus, Phalloidin (red) for F-actin, Villin (magneta) for enterocytes, and Chromogranin A (orange) for enteroendocrines. Scale bar, 20 µM.

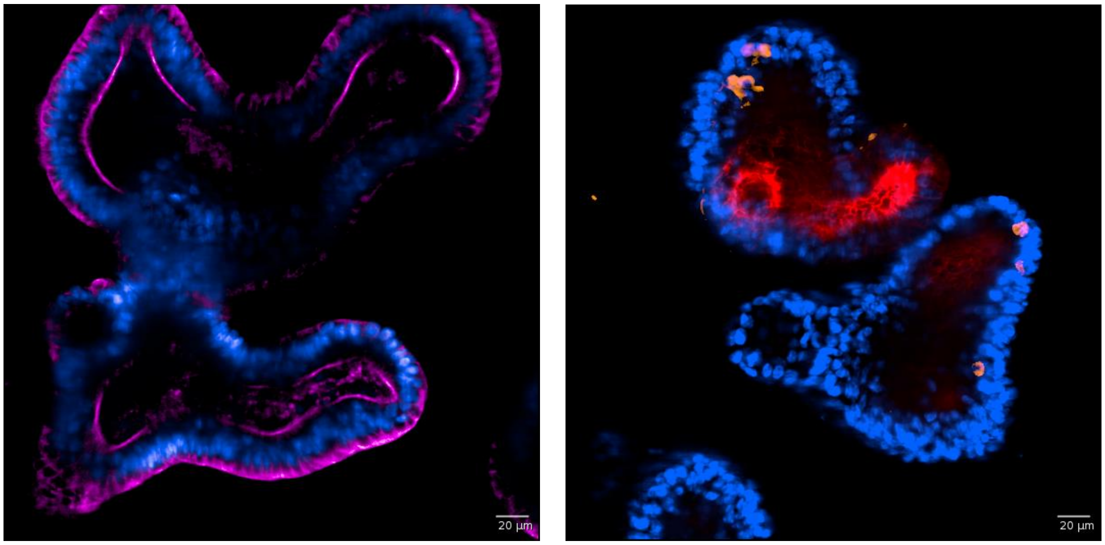

DAPI Villin

DAPI Phalloidin Chromogranin A

Supplemental Figure S2. EV-A71 replication kinetics in RD cells.

(A) EV-A71 replication kinetics. RD cells were infected with EV-A71 virus at 0.01, 0.1, and 1 MOI. At the indicated time points, the levels of total extracellular viral RNA were measured by RT-qPCR. (**B**) Viability of RD cells post EV-A71 infection. To measure viability of RD cells, Cell Titer-Glo was used at the indicated time points post infection. Mock infected cells were used to normalize the viability as a percentage. Data are the mean ± SEM of three independent experiments; each carried out in triplicate. * indicates p < 0.05; ** indicates p < 0.01; *** indicates p < 0.001; **** indicates p < 0.0001; ns indicates not significant.

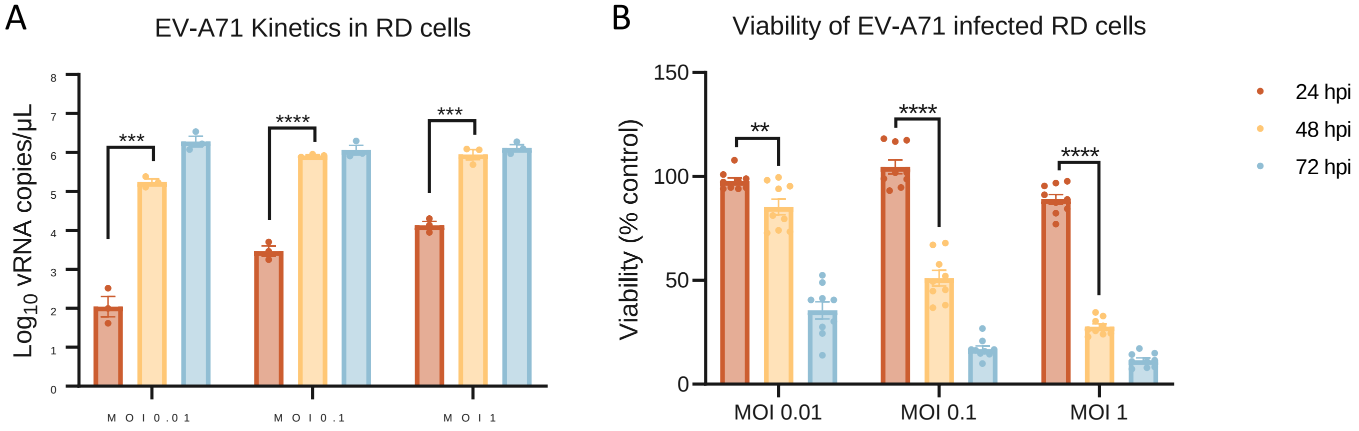

Supplemental Figure S3. Brightfield images of HIOs and RD cells.

Mock control, EV-A71 infection (0.1 MOI) treated with 1.25 µM of enviroxime, rupintrivir, or 2'CMC, and EV-A71 infection control (0.1 MOI) of HIOs and RD cells at 72 hpi. All images were acquired using the same objective. Scale bar, 100 µM.

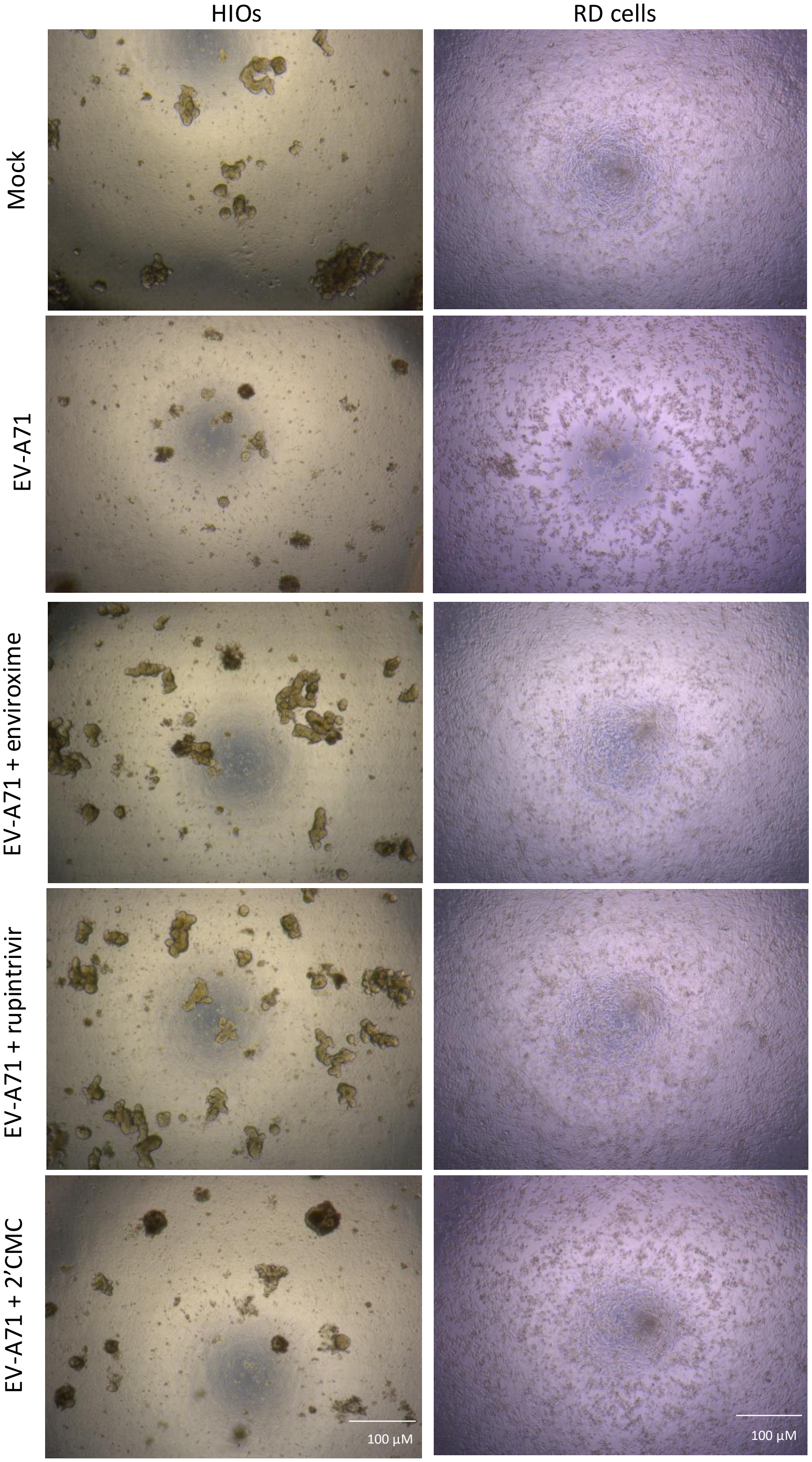

Supplemental Figure S4. Immunofluorescence images of HIOs.

Images represent mock, EV-A71 infected HIOs (0.1 MOI), EV-A71 infected HIOs (0.1 MOI) treated with rupintrivir (10 µM), and EV-A71 infected HIOs (0.1 MOI) treated with 2'CMC (50 µM) at 48 hpi: DAPI (blue) for nucleus, Phalloidin (red) for F-actin, and dsRNA (green). Scale bar, 100 µM.

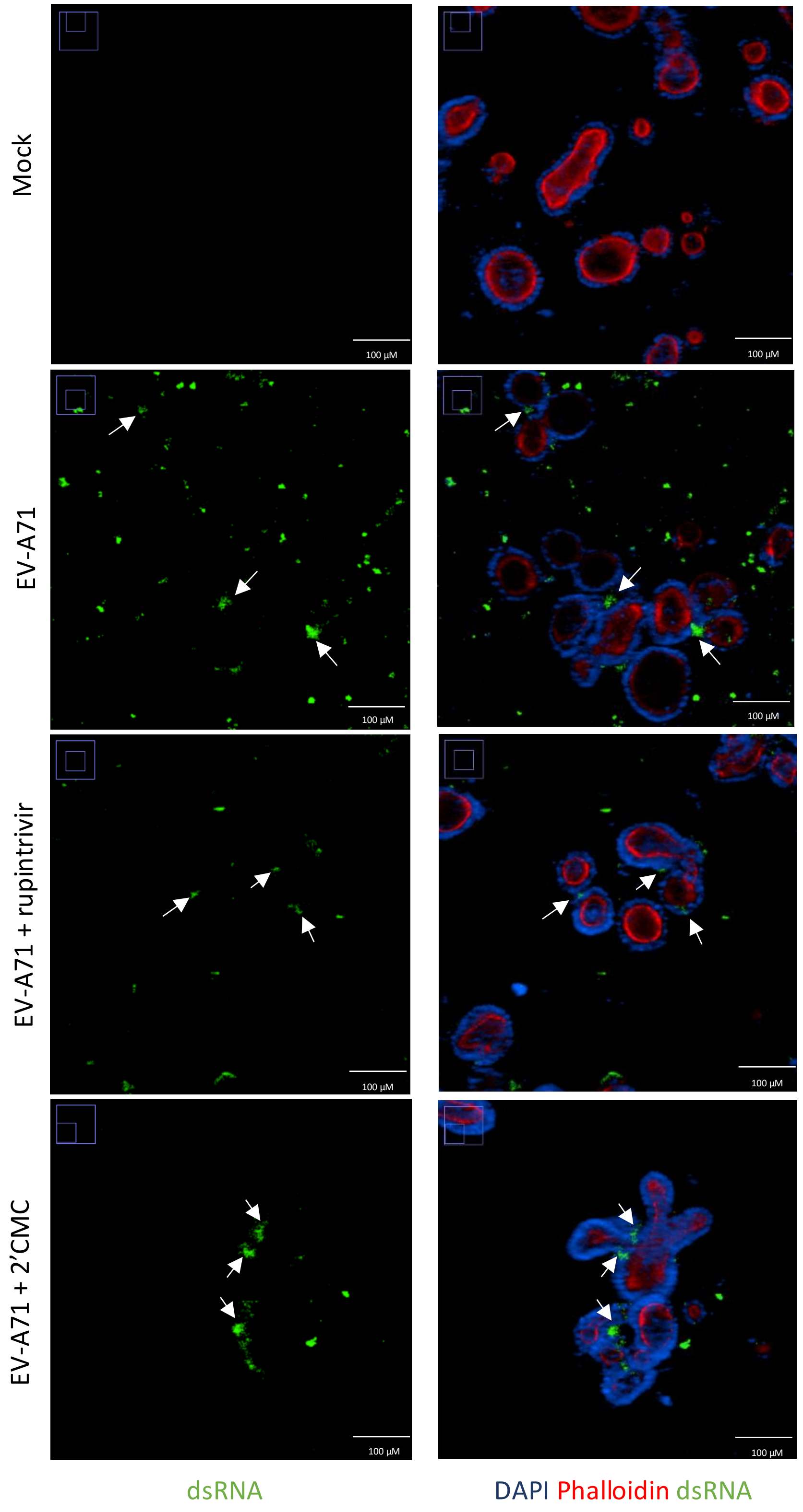

Supplemental Figure S5. Immunofluorescence images of RD cells.

Images represent EV-A71 infected RD cells (0.1 MOI), EV-A71 infected HIOs (0.1 MOI) treated with different concentrations of rupintrivir or 2’CMC at 24 hpi: DAPI (blue) for nucleus, Phalloidin (red) for F-actin, and dsRNA (green). Scale bar, 311.6 µM.

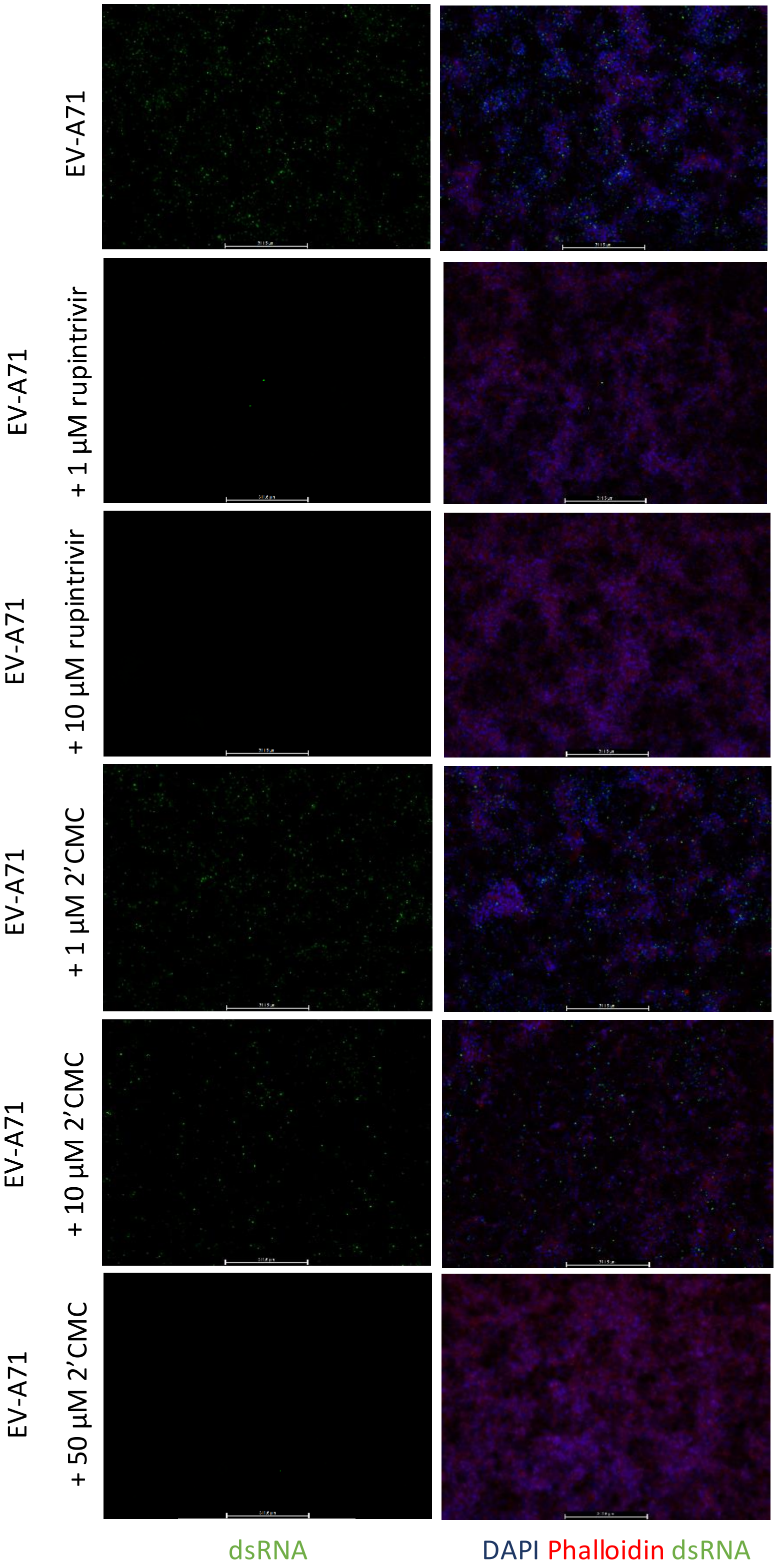

Supplement: Supplementary file 1 [file cells-12-01138-s001.zip › cells-2199218-supplementary.pdf]
